# Therapeutic Potential of Tanshinones in Osteolytic Diseases: From Molecular and Cellular Pathways to Preclinical Models

**DOI:** 10.3390/dj13070309

**Published:** 2025-07-09

**Authors:** Rafael Scaf de Molon

**Affiliations:** Department of Diagnosis and Surgery, School of Dentistry at Araçatuba, São Paulo State University, Araçatuba 16015-050, Brazil; rafael.molon@unesp.br; Tel.: +55-16-997309293 or +55-18-36362860

**Keywords:** alveolar bone loss, histology, osteoclasts, periodontal disease, tanshinone II A sodium sulfonate, tanshinone IIa

## Abstract

Tanshinones are a class of lipophilic diterpenoid quinones extracted from *Salvia miltiorrhiza* (Dan shen), a widely used herb in traditional Chinese medicine. These compounds, particularly tanshinone IIA (T-IIA) and sodium tanshinone sulfonate (STS), have been acknowledged for their broad spectrum of biological activities, including anti-inflammatory, antioxidant, anti-tumor, antiresorptive, and antimicrobial effects. Recent studies have highlighted the potential of tanshinones in the treatment of osteolytic diseases, characterized by excessive bone resorption, such as osteoporosis, rheumatoid arthritis, and periodontitis. The therapeutic effects of tanshinones in these diseases are primarily attributed to their ability to inhibit osteoclast differentiation and activity, suppress inflammatory cytokine production (e.g., tumor necrosis factor alpha (TNF-α), interleukin (IL)-1β, and IL-6), and modulate critical signaling pathways, including NF-kB, MAPK, PI3K/Akt, and the RANKL/RANK/OPG axis. Additionally, tanshinones promote osteoblast differentiation and mineralization by enhancing the expression of osteogenic markers such as Runx2, ALP, and OCN. Preclinical models have demonstrated that T-IIA and STS can significantly reduce bone destruction and inflammatory cell infiltration in arthritic joints and periodontal tissues while also enhancing bone microarchitecture in osteoporotic conditions. This review aims to provide a comprehensive overview of the pharmacological actions of tanshinones in osteolytic diseases, summarizing current experimental findings, elucidating underlying molecular mechanisms, and discussing the challenges and future directions for their clinical application as novel therapeutic agents in bone-related disorders, especially periodontitis. Despite promising in vitro and in vivo findings, clinical evidence remains limited, and further investigations are necessary to validate the efficacy, safety, and pharmacokinetics of tanshinones in human populations.

## 1. Introduction

Osteolytic disorders such as osteoporosis, rheumatoid arthritis, periodontal disease, cancer-induced bone disease, and diabetic osteopathy are linked by a pathological excess of osteoclastic bone resorption, which outpaces osteoblastic bone formation [1,2,3,4]. The clinical and societal burden of these conditions is considerable. For instance, osteoporosis alone endangers an estimated 200 million women worldwide and contributes to roughly 37 million fragility fractures annually, with one in three women and one in five men over the age of fifty expected to experience an osteoporotic fracture during their lifetime [5,6]. Periodontal diseases, especially periodontitis, accounted for nearly one billion prevalent cases and 6.2 million disability-adjusted life-years in 2021 [7]. Current therapeutic options, such as bisphosphonates, denosumab, cathepsin K inhibitors, non-steroidal anti-inflammatory drugs, and biologic disease-modifying antirheumatic drugs, do mitigate fracture or erosion risk. However, these medications are hindered by adverse-event profiles that include osteonecrosis of the jaw [8,9], rebound vertebral fractures, and systemic immunosuppression [10,11]. These limitations have gained interest in naturally derived, pleiotropic small molecules capable of coupling anti-inflammatory activity with direct inhibition of osteoclastogenesis while preserving, or even stimulating, osteoblast function [12,13,14,15].

**Figure 1:**
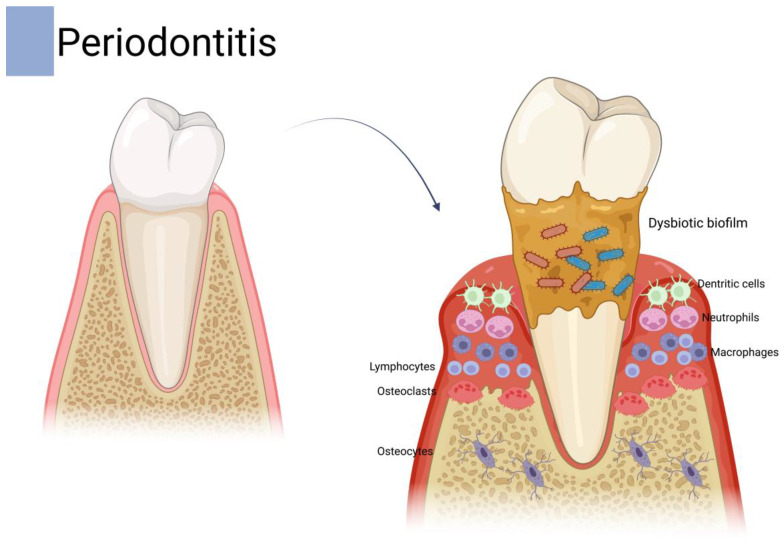
Transition from health to disease during periodontitis pathogenesis.

Periodontitis is a chronic, multifactorial inflammatory disease that compromises all periodontal tissues, including the gingiva, periodontal ligament, cementum, and alveolar bone, ultimately jeopardizing tooth support and oral function. Its initiation hinges on the formation of a complex polymicrobial biofilm around the enamel or cement close to the gingival margin and within the periodontal pocket [16,17]. In health, a state of immunological homeostasis is sustained: the resident microbiota supplies benign stimuli that stimulate innate and adaptive immune cells, while host surveillance mechanisms confine microbial proliferation and prevent collateral tissue injury. Disease emerges when this finely tuned equilibrium shifts toward dysbiosis (Figure 1) [18]. A prevailing hypothesis posits that colonization by so-called “keystone” pathogens, exemplified by *Porphyromonas gingivalis (P. gingivalis)*, can disproportionately remodel the microbial community despite their low abundance [19]. *P. gingivalis* interferes with complement–TLR crosstalk, dampens neutrophil bactericidal activity, and alters nutrient availability in ways that favor the outgrowth of proteolytic, pro-inflammatory species [19]. The resulting ecological transition magnifies the biofilm’s pathogenicity and provokes a persistent host response.

The host inflammatory cascade is characterized by a stream of neutrophils, macrophages, and dendritic cells that secrete interleukin (IL)-1β, tumor necrosis factor-α (TNF-α), IL-6, and prostaglandin E_2_ (PGE2). These mediators, together with matrix metalloproteinases (MMP) and reactive oxygen species (ROS), orchestrate soft-tissue degradation. Concurrently, antigen presentation activates T and B cells, driving a Th17-skewed adaptive response and robust expression of receptor activator of nuclear factor-κB ligand (RANKL) [20]. Elevated RANKL, opposed by insufficient osteoprotegerin (OPG), promotes osteoclastogenesis and accelerates alveolar bone resorption, the radiographic hallmark of periodontitis. Sustained inflammation therefore perpetuates a vicious cycle of microbial overgrowth, immune activation, and progressive loss of periodontal attachment apparatus [16]. Importantly, periodontitis has been linked to several non-communicable chronic inflammatory conditions, such as diabetes [21,22,23], rheumatoid arthritis [24,25,26,27], chronic kidney disease [28], and cardiovascular diseases [29,30,31,32], making its treatment of utmost importance.

According to the European Federation of Periodontology (EFP) [33], the treatment of periodontitis follows a structured, evidence-based, and individualized approach comprising four sequential and interdependent phases. The first phase, initial therapy, aims to control the underlying etiological factors through comprehensive patient education, behavior modification, particularly improvements in oral hygiene and smoking cessation, and professional mechanical plaque removal. Following supragingival biofilm control, subgingival instrumentation (SI) is performed as a second step to mechanically disrupt and remove subgingival biofilms and calculus deposits. Although this non-surgical approach is generally effective in reducing inflammation and halting disease progression, a subset of patients may continue to exhibit persistent periodontal pockets, typically defined as sites with probing depths ≥4 mm and bleeding on probing, which signal the need for further therapeutic intervention [33]. In such cases, re-instrumentation and additional non-surgical therapy may be indicated, potentially in combination with adjunctive treatments, such as locally delivered antimicrobials [34] or host-modulating agents, to enhance the healing response and improve clinical outcomes [33]. The third phase, or corrective therapy, involves periodontal surgery, which is reserved for residual pockets unresponsive to non-surgical or adjunctive therapies. This may include surgical access for debridement or regenerative procedures aimed at restoring lost periodontal structures [33]. The fourth phase, supportive periodontal care, constitutes a lifelong maintenance protocol designed to prevent recurrence of disease through regular clinical monitoring, reinforcement of oral hygiene practices, and periodic professional debridement. Throughout all phases, active patient engagement remains a cornerstone of successful therapy, as sustained behavioral change is essential for achieving and maintaining periodontal health. This systematic, stepwise approach ensures that care is tailored to the individual patient’s needs and adapted to the severity and complexity of the disease.

Adjuvant therapies have emerged as valuable complements to conventional periodontal treatment, particularly in cases where standard mechanical debridement alone yields suboptimal outcomes. A growing body of evidence from both animal models and clinical studies supports the use of natural compounds with anti-inflammatory, antioxidant, antimicrobial, and bone-protective properties as effective adjuncts in the management of periodontitis [12,15,35,36,37,38,39,40]. These agents, including polyphenols (curcumin and resveratrol) [36,38,40], flavonoids (quercetin and luteolin) [41,42,43], cystatins [39], and terpenoids (tanshinone IIA) [44], have demonstrated the ability to modulate key inflammatory pathways such as NF-κB, MAPK, and NLRP3 inflammasome signaling. In preclinical models, these compounds have been shown to reduce inflammatory cell infiltration, suppress pro-inflammatory cytokine expression, and attenuate alveolar bone loss [36,38,39,40,41,42,43]. Clinically, adjunctive use of natural compounds, whether applied locally (e.g., gels, chips, or mouth rinses) or systemically, has been associated with improvements in clinical parameters such as probing pocket depth reduction, clinical attachment gain, and bleeding on probing [35,36,37]. Furthermore, their favorable safety profiles and potential to promote tissue regeneration underscore their relevance in integrative periodontal therapy. The current literature suggests that natural compound-based adjuvant therapies offer a promising and biologically sound strategy to enhance periodontal treatment outcomes, particularly in patients with persistent inflammation or systemic comorbidities.

Osteolytic diseases pose a significant therapeutic dilemma due to the complexity of their pathogenesis and the limited efficacy and potential side effects of current treatment options such as bisphosphonates and monoclonal antibodies. These osteolytic conditions, including periodontitis, often involve chronic inflammation and an imbalance in bone remodeling, leading to progressive bone loss and structural deterioration. Recent research studies have highlighted the therapeutic potential of tanshinones, a group of bioactive diterpenoids derived from *Salvia miltiorrhiza*, which exhibit notable biological properties [45,46,47]. Tanshinones have demonstrated promising effects in preclinical models of osteolytic diseases by modulating key signaling pathways such as NF-κB, MAPK, and RANKL/OPG, thereby suppressing osteoclastogenesis and mitigating inflammation-induced bone destruction [45,46,47]. These findings suggest that tanshinones may offer a novel and multifaceted approach to managing osteolytic diseases, particularly where inflammation and bone resorption converge, warranting further exploration in translational and clinical settings [48].

The aim of this narrative review was to describe the possible role of tanshinone, a natural compound, in the management of osteolytic disease, with emphasis on its pharmacological actions, summarizing current experimental findings, elucidating underlying molecular mechanisms, and discussing the challenges and future directions for their clinical application as novel therapeutic agents in bone-related disorders, especially periodontitis.

## 2. Tanshinones

The diterpenoid quinones collectively known as tanshinones, lipophilic constituents isolated from the roots of *Salvia miltiorrhiza*, commonly referred to as Dan Shen in traditional Chinese medicine, have garnered increasing scientific interest due to their diverse pharmacological properties [13,14,49]. Based on their chemical structures and solubility profiles, these bioactive compounds are generally classified into two major groups: phenolic acids and abietane-type diterpene quinones [46,50]. Phenolic acids are hydrophilic (water-soluble) molecules, with salvianolic acid being a representative and widely studied example. In contrast, the abietane-type diterpene quinones are lipophilic (fat-soluble) and include key compounds such as cryptotanshinone (CTS) and tanshinone IIA (T-IIA) [46,50].

To date, more than 40 diterpene quinones and over 30 phenolic acids have been identified in *Salvia miltiorrhiza*. Among the diterpene quinones, notable compounds with strong pharmacological activities include tanshinone I (T-I), dihydrotanshinone I (DTI), T-IIA, tanshinone IIB, tanshilactone, and CTS [46,51,52] (Table 1). Within the phenolic acid group, the primary bioactive constituents comprise salvianolic acid A, salvianolic acid B, salvianolic acid C, and lithospermic acid.

**Figure 2:**
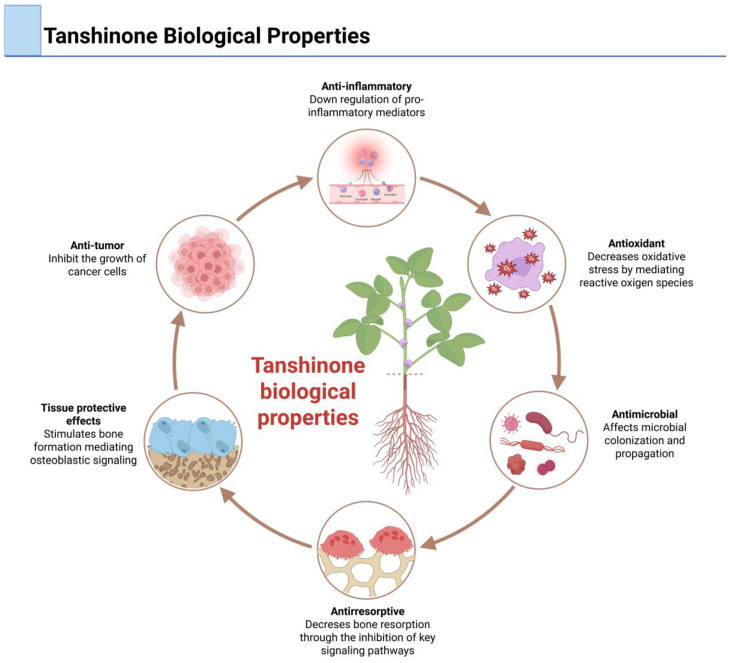
Biological properties of tanshinones including anti-inflammatory, antioxidant, antimicrobial, antiresorptive, tissue-protective, and anti-tumor effects.

Current evidence demonstrates that these constituents contribute to a range of therapeutic effects, including antioxidant, anti-inflammatory, antimicrobial, antiresorptive, anti-tumor, and tissue-protective effects, which may have implications for the management of chronic inflammatory osteolytic diseases [46,51,52] (Figure 2). Beyond their well-documented biological properties, tanshinones have demonstrated the capacity to modulate bone and immune cell behavior in a manner that addresses the composite pathophysiology of osteolytic diseases [13,14,46,49,53,54,55,56].

**Figure 3:**
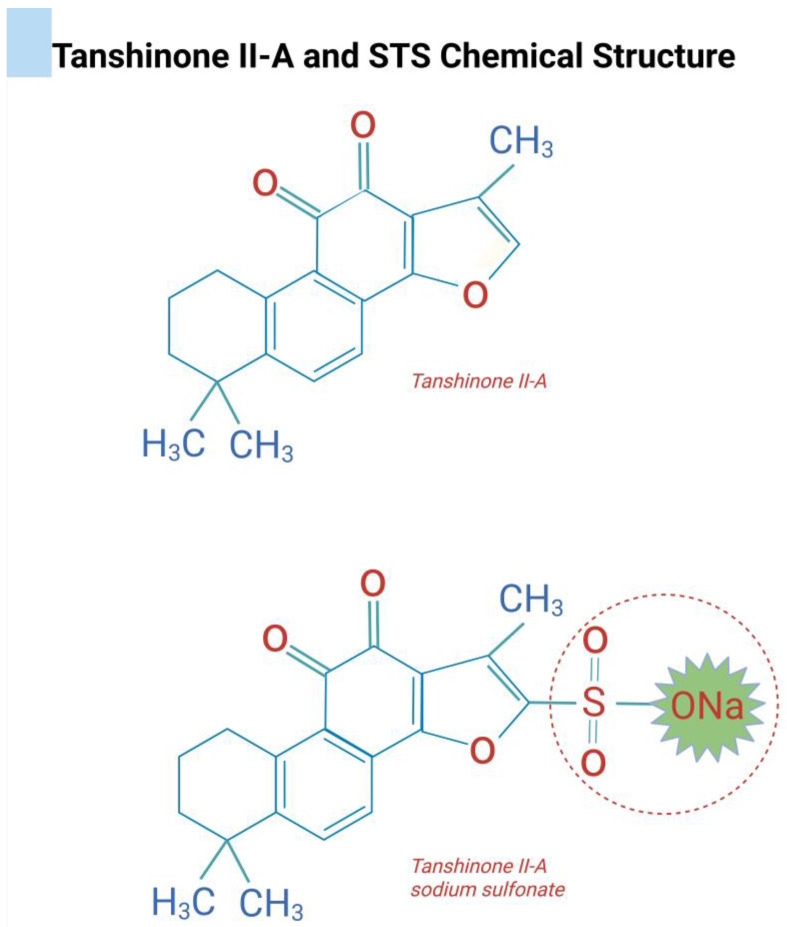
The diterpenoid quinones collectively known as tanshinones are lipophilic constituents isolated from the roots of *Salvia miltiorrhiza*, commonly referred to as Dan Shen. Representative chemical structure of both T-IIA and STS, respectively.

Tanshinones are characterized by a distinctive tetracyclic structure composed of four fused rings (Figure 3). The core scaffold typically includes rings A and B, which form a naphthalene or tetrahydronaphthalene system [46]. The biological activity of tanshinones is closely associated with their unique terpenoid and quinone-based chemical structure, which supports both cellular permeability and specific molecular interactions [14,45,47,49,52,53]. The lipophilic terpenoid structure of tanshinones facilitates efficient transmembrane diffusion, allowing them to readily cross cell membranes and access intracellular targets. Once inside the cell, tanshinones offer multiple binding interfaces for key signaling proteins and enzymes, enabling precise modulation of intracellular pathways. Additionally, the quinone fraction functions as an electron carrier in redox reactions, influencing oxidative signaling cascades and contributing to the broad pharmacological activities of these compounds [46,51,52]. These properties have long been exploited in the treatment of experimental rheumatoid arthritis [54], cardiovascular diseases [57,58,59,60], cancer [51,61,62,63,64], diabetes mellitus [65], intestinal inflammation [66,67,68], inflammatory processes [69], and periodontal disease [44].

**Figure 4:**
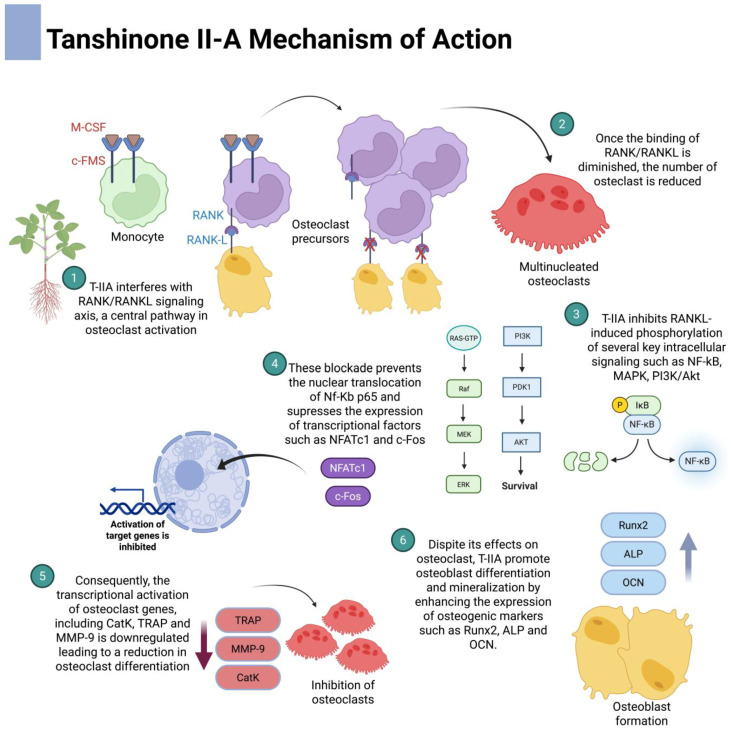
Mechanism of action of tanshinone on bone. Tanshinone inhibits osteoclast formation and differentiation by suppressing key signaling pathways, which leads to the downregulation of transcription factors and a subsequent decrease in the expression of target genes. P means phosphorylation.

T-IIA, one of the most extensively studied tanshinone derivatives, demonstrates potent activity against pathological bone resorption through the modulation of osteoclastogenesis [54,55,56,70]. At the molecular level, T-IIA interferes with the RANK-RANKL signaling axis, a central pathway in osteoclast differentiation and activation. In vitro studies consistently show that T-IIA, along with other derivatives such as T-I and DTI, inhibits RANKL-induced phosphorylation of several key intracellular signaling molecules, including NF-κB, ERK, p38 MAPK, JNK, and Akt [71,72,73]. This blockade prevents the nuclear translocation of NF-κB p65 and suppresses the expression of essential osteoclastogenic transcription factors such as c-Fos and NFATc1 [74]. Consequently, the transcriptional activation of osteoclast-specific genes, including tartrate-resistant acid phosphatase (TRAP), cathepsin K, the calcitonin receptor, and MMP-9, is significantly downregulated, leading to a marked reduction in osteoclast differentiation and resorptive activity (Figure 4) [74].

Redox regulation provides an additional layer of osteoclast restraint. T-IIA covalently modifies lactate-dehydrogenase-C, lowering intracellular ROS and blunting ROS-driven osteoclastogenesis in experimental arthritis [75], while CTS and TI have similarly been shown to neutralize oxidative stress in osteoclast precursors. Concomitantly, tanshinones foster osteogenesis: T-IIA activates ERK1/2–Runx2 signaling, up-regulates phosphoglycerate dehydrogenase, and rejuvenates senescent bone-marrow mesenchymal stem cells, thereby promoting osteoblastic differentiation, matrix deposition, and mineralization [76]. Their immunomodulatory influence is equally compelling; some studies document inhibition of TLR4/MyD88 and NLRP3 inflammasome activation, down-regulation of IL-1β, TNF-α, and IL-17, and tempering of innate and adaptive immune-cell infiltration into inflamed tissues [54]. By converging on inflammatory, oxidative, and catabolic pathways, tanshinones offer a coherent pharmacological solution to the intertwined drivers of bone destruction.

T-IIA exhibits limited oral bioavailability due to its high hydrophobicity. To address this pharmacokinetic limitation, a sulfonated derivative, sodium tanshinone IIA sulfonate (STS), was synthesized, significantly enhancing its water solubility and oral bioavailability [46,54,55,56]. One of the pioneering studies characterizing STS was conducted by Panwar et al. (2018), which systematically evaluated several tanshinone compounds for their ability to inhibit cathepsin K, a cysteine protease pivotal to osteoclastic bone resorption [55]. Among 31 tested compounds, 12 displayed potent anti-collagenase activity, and 6 notably suppressed osteoclastic bone resorption without adversely affecting osteoclast differentiation, viability, or transforming growth factor-β1 (TGF-β1) degradation [55]. Importantly, STS emerged as a highly specific and effective inhibitor, preserving the mechanical integrity of collagen fibers and demonstrating a safer profile compared to traditional cathepsin K inhibitors.

**Figure 5:**
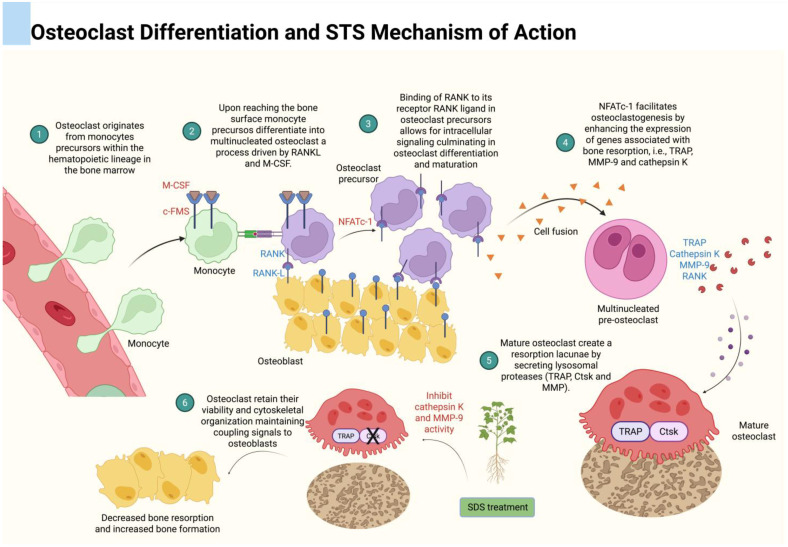
STS directly inhibits cathepsin K enzymatic activity by binding to an ectosteric site located near the enzyme’s collagen-binding exosite. This non-competitive mechanism disrupts the spatial alignment of the triple-helical collagen substrate within the enzyme’s active site, selectively inhibiting its collagenolytic activity while sparing general endopeptidase function. Such selective interference allows for the preservation of cathepsin K’s catalytic architecture, avoiding compensatory protease overexpression or osteoclast apoptosis. This mode of action allows osteoclasts to retain their viability and cytoskeletal organization, maintaining their coupling signals to osteoblasts, which are essential for bone remodeling. “×” means blockage of cathepsin K expression.

STS is synthesized via sulfonation of T-IIA at the carbon-16 position, yielding a compound with the molecular formula C_19_H_17Na_O_6S_, in contrast to the compound T-IIA (C_19_H_18_O_3_) [55] (Figure 3). This structural modification not only improves aqueous solubility but also alters the compound’s pharmacokinetic and pharmacodynamic properties. Unlike T-IIA, which predominantly targets upstream signaling pathways in osteoclastogenesis, STS directly inhibits cathepsin K enzymatic activity by binding to an ectosteric site located near the enzyme’s collagen-binding exosite [13,46] (Figure 5). This non-competitive mechanism disrupts the spatial alignment of the triple-helical collagen substrate within the enzyme’s active site, selectively inhibiting its collagenolytic activity while sparing general endopeptidase function. Such selective interference allows for the preservation of cathepsin K’s catalytic architecture, avoiding compensatory protease overexpression or osteoclast apoptosis, adverse outcomes frequently associated with conventional active-site-directed inhibitors [13,46].

Importantly, this mode of ectosteric inhibition allows osteoclasts to retain their viability and cytoskeletal organization, thereby maintaining their coupling signals to osteoblasts, which are essential for balanced bone remodeling [55,56,70] (Figure 5). The selective attenuation of collagenolysis without compromising bone formation distinguishes STS from earlier-generation cathepsin K inhibitors like odanacatib, which were associated with impaired bone remodeling and adverse cardiovascular events [77]. By preserving osteoclast function and avoiding rebound resorption, STS represents a novel paradigm in the modulation of proteolytic enzymes through ectosteric inhibition and offers a promising therapeutic strategy for skeletal disorders characterized by excessive bone degradation, such as periodontitis [54,55].

## 3. Tanshinone and Osteoclast Molecular Pathways

Osteoclasts are specialized, multinucleated cells responsible for the resorption of bone matrix during normal bone remodeling and pathological bone loss. These cells originate from hematopoietic precursors of the monocyte/macrophage lineage within the bone marrow microenvironment [78] (Figure 5). A network of signaling pathways, with two cytokines playing a central role, i.e., macrophage colony-stimulating factor (M-CSF) and RANKL, tightly regulates their differentiation, proliferation, and activation.

M-CSF promotes the survival and proliferation of osteoclast precursors by binding to its receptor, c-Fms, leading to downstream activation of signaling molecules such as PI3K/Akt and ERK [79,80,81,82]. RANKL, expressed by osteoblasts, osteocytes, and stromal cells, interacts with its receptor RANK on osteoclast precursors, initiating a complex cascade involving the recruitment of the adaptor protein TRAF6 and activation of multiple downstream pathways [4,80,83]. These include the NF-κB pathway, MAPK cascades (ERK, JNK, and p38), and calcium signaling, all of which converge on key transcription factors such as c-Fos and nuclear factor of activated T cells 1 (NFATc1). NFATc1 serves as the master regulator of osteoclastogenesis, controlling the transcription of osteoclast-specific genes such as TRAP, cathepsin K, calcitonin receptor, and MMP-9 [84]. Dysregulation of this signaling axis leads to excessive osteoclast formation and heightened bone resorption, contributing to the pathophysiology of conditions such as osteoporosis, rheumatoid arthritis, and periodontitis.

**Figure 6:**
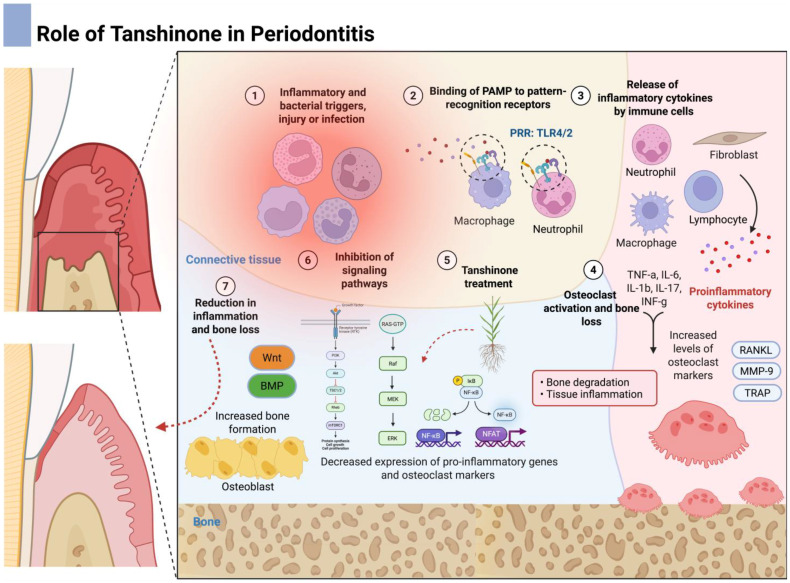
Role of tanshinone in osteolytic diseases. A representative scheme illustrating bone destruction amelioration through the inhibition of key signaling pathways, including MAPK, NF-kB, and AKT. Moreover, increased bone formation might also be related to tanshinone treatment due to the effects on osteoblastic signaling (Wnt and BMP pathways).

Tanshinones have been shown to exert significant regulatory effects on osteoclast differentiation and function by targeting critical signaling pathways, as described earlier [71,72,73]. As a result, the expression of key osteoclastic markers involved in bone matrix degradation is downregulated (Figure 6).

Kwak et al. (2006) investigate the molecular mechanisms by which T-IIA suppresses osteoclastogenesis [85]. This work demonstrates that T-IIA significantly inhibits RANKL-induced differentiation of osteoclast precursors without affecting the expression of RANK or c-Fms, indicating that its action targets downstream signaling. Specifically, T-IIA blocks the expression of the transcription factors c-Fos and NFATc1, both essential for osteoclast formation. Overexpression of c-Fos or NFATc1 via retroviral transduction reverses the inhibitory effects of T-IIA, confirming their role as key mediators [85]. The findings suggest that T-IIA suppresses osteoclastogenesis by disrupting RANKL-mediated activation of c-Fos, which in turn impairs NFATc1 induction [85].

Panwar et al. (2016) explores the use of DT1 as a selective exosite inhibitor of cathepsin K [70]. Unlike conventional active-site inhibitors such as odanacatib, which indiscriminately inhibit all catalytic functions of cathepsin K and are associated with off-target effects, DT1 selectively interferes with cathepsin K collagenase activity by binding to a non-catalytic ectosteric site. This targeted mechanism disrupts the enzyme’s ability to degrade collagen fibers while sparing its general endopeptidase functions, thereby preserving osteoclast viability and minimizing adverse effects such as tissue fibrosis, which are frequently observed with active-site inhibitors [70]. This study demonstrated that DT1 significantly attenuates bone resorption without altering osteoclast number or their resorptive activity on non-collagenous substrates [70]. These findings support the therapeutic promise of ectosteric inhibitors like DHT1 and STS in the treatment of osteoporosis and other bone-resorptive disorders.

### 3.1. Nuclear-Factor-κB (NF-κB) Signaling

NF-κB is a pivotal transcription factor that connects inflammatory danger signals to the gene program driving osteoclastogenesis, matrix catabolism, and the metabolic reprogramming of immune and stromal cells [86]. Canonically, the engagement of pattern-recognition receptors, such as Toll-like receptor 4 (TLR4) or members of the TNF receptor superfamily, notably RANK, recruits the TRAF6–TAK1 complex. This activation triggers the IκB kinase (IKK) holoenzyme, leading to the proteasomal degradation of cytoplasmic IκBα. As a result, p65/p50 dimers are released and translocated into the nucleus to initiate transcription [87]. In osteoclast precursors, a parallel non-canonical arm, centered on NF-κB-inducing kinase (NIK) and IKKα, generates p52/RelB heterodimers that cooperate with c-Fos and NFATc1 to complete the differentiation program [88]. Tanshinone intercepts this cascade at multiple hierarchical levels, conferring a broad yet convergent anti-inflammatory and anti-resorptive signature.

A defining study using a purified total tanshinones, composed of 17% of T-IIA and 8% of cryptotanshinone, showed that the mixture hinders LPS-induced TLR4 homodimerization, thereby curtailing recruitment of MyD88 and abrogating TAK1 phosphorylation [89]. The result is rigorous suppression of IKKβ and IκBα phosphorylation, diminished p65 nuclear translocation, and blunted transcriptional activity of both NF-κB and AP-1 in macrophages and bone marrow-derived monocytes (BMDM) [89].

A previous study, using the RAW-264.7 macrophage cell line, demonstrates that T-IIA dose-dependently suppresses phosphorylation of NIK and both catalytic subunits of the IKK complex, prevents IκBα degradation, and ultimately inhibits p65/p50 DNA-binding activity [90]. CTS reproduces this pattern: it abolishes LPS-triggered IκBα and IKK phosphorylation, blocks p65 nuclear translocation, and concomitantly decreases ERK, p38, and JNK activation, highlighting extensive crosstalk between NF-κB and MAPK signaling [91]. STS also dampens NF-κB phosphorylation and nuclear accumulation across cardiovascular, hepatic, and vascular models [13].

In summary, tanshinone derivatives act as multi-level modulators of the NF-κB signaling pathway. They interfere with receptor complex assembly at upstream sites such as TLR4 and RANK, suppress downstream kinase signaling cascades including the NIK–IKK axis, and stabilize cytoplasmic inhibitors like IκBα to prevent nuclear translocation of NF-κB. Additionally, through redox-sensitive mechanisms, tanshinones suppress oxidative stress-induced feedback loops that sustain chronic inflammation and osteolysis. This broad-spectrum yet coordinated inhibition of NF-κB signaling provides a molecular basis for their therapeutic.

### 3.2. Mitogen-Activated Protein Kinase (MAPK) Signaling

The MAPK signaling cascade constitutes a critical factor in the regulation of osteoclast differentiation, inflammatory cytokine production, stress response, and apoptosis [86]. It comprises three principal arms: extracellular signal-regulated kinases (ERK1/2), c-Jun N-terminal kinases (JNK), and p38 MAPKs, each activated through a conserved three-tier phosphorylation module in response to various extracellular stimuli, including RANKL, LPS, and pro-inflammatory cytokines [86]. These kinases function cooperatively and differentially to modulate the activation of downstream transcription factors such as c-Fos, AP-1, and NFATc1, which are essential for osteoclastogenesis, as well as to mediate the expression of inflammatory mediators including TNF-α, IL-1β, and COX-2 in macrophages and other immune cells. Tanshinones have been shown to exert robust inhibitory effects on the MAPK signaling pathways, thereby modulating inflammation and bone resorption through direct interference with kinase activation and transcriptional output [46,74,91].

Experimental evidence indicates that tanshinones suppress MAPK signaling primarily at the level of upstream MAPK kinases (MAP2Ks), leading to decreased phosphorylation of ERK1/2, JNK, and p38 [74,89]. In RANKL-stimulated bone marrow–derived macrophages (BMMs), treatment with T-IIA significantly reduces the phosphorylation of MEK1/2 and ERK1/2 within 15–30 min of exposure, thus inhibiting the activation of c-Fos, a component of the AP-1 complex required for NFATc1 expression and full osteoclast differentiation [72,74]. Similar attenuation is observed for p38 and JNK signaling arms, with downstream suppression of ATF2 and c-Jun phosphorylation. The result is a profound inhibition of osteoclast-specific gene expression [72,74,91]. Notably, CTS has been shown to exert comparable effects, abrogating LPS- or RANKL-induced MAPK phosphorylation and limiting NFATc1 accumulation, thus curtailing osteoclastogenesis and bone resorption both in vitro and in vivo [91].

Mechanistically, the precise binding targets of tanshinones within the MAPK axis remain under investigation, but molecular docking and kinase assays suggest that these compounds may act as ATP-competitive inhibitors or interfere with kinase–scaffold interactions. Additionally, the antioxidant properties of T-IIA and CTS, such as reduction in intracellular ROS, may contribute indirectly to MAPK inhibition, as oxidative stress is a known activator of MAPK cascades, particularly JNK and p38.

### 3.3. Phosphoinositide 3-Kinase PI3K/Akt Signaling Pathway

The PI3K/Akt signaling pathway is a central regulator of cell survival, proliferation, differentiation, and metabolism [92]. PI3K/Akt signaling plays a pivotal role in osteoclastogenesis, osteoblast survival, and immune cell activation, and its dysregulation contributes to the pathogenesis of inflammatory and osteolytic diseases [93]. Activation of the PI3K/Akt pathway is typically initiated by receptor engagement, including RANKL, TNF-α, or TLR, which leads to the generation of phosphatidylinositol (3,4,5)-trisphosphate (PIP3) and subsequent phosphorylation and activation of Akt (protein kinase B). Activated Akt transduces signals to multiple downstream effectors, including mTOR, NF-κB, and FOXO1 transcription factors, which coordinate the expression of genes involved in cell survival, osteoclast differentiation, matrix degradation, and inflammation [93].

In the study by Chiu et al. (2009), T-IIA was identified as an effective inhibitor of the PI3K/Akt/AP-1 signaling axis in human synovial fibroblasts stimulated by peptidoglycan (PGN), a component of Gram-positive bacterial cell walls [94]. PGN was shown to increase IL-6 production via activation of TLR2, followed by the sequential activation of focal adhesion kinase (FAK), PI3K, and Akt, ultimately leading to nuclear translocation and activation of c-Jun/AP-1, a transcription factor critical for IL-6 gene expression. Pretreatment with T-IIA significantly suppressed PGN-induced IL-6 secretion and reduced AP-1 transcriptional activity, implicating AP-1 as the downstream target of this regulatory cascade. These findings indicate that T-IIA exerts anti-inflammatory effects by disrupting Akt-dependent c-Jun phosphorylation and AP-1 activation, positioning it as a promising modulator of inflammation in rheumatoid arthritis and related conditions [94].

Importantly, tanshinones also appear to influence the osteoblastic arm of bone remodeling through the PI3K/Akt pathway [95]. In osteoblast-like MC3T3-E1 cells and mesenchymal stem cells (MSCs), low concentrations of T-IIA have been shown to modestly activate PI3K/Akt signaling, promoting cell survival and enhancing Runx2-mediated osteogenic differentiation under oxidative or inflammatory stress [96]. This bidirectional regulatory behavior suggests that tanshinones may exert context-dependent effects, suppressing PI3K/Akt signaling in osteoclasts and immune cells while supporting anabolic processes in osteoblasts, particularly under pathologic conditions.

### 3.4. RANK/RANKL/OPG Axis

RANK–RANKL–OPG triad orchestrates the balance between bone resorption and formation [4,80,83]. RANKL, produced mainly by osteoblast–osteocyte lineage and immune cells, binds its cognate receptor RANK on osteoclast precursors to trigger NF-κB, MAPK, and PI3K/Akt cascades, culminating in NFATc1-driven osteoclastogenesis and matrix degradation [4,80,83]. OPG counters this process as a soluble decoy receptor that sequesters RANKL and terminates signaling [97,98]. Disruption of the RANKL/OPG ratio therefore skews bone turnover toward pathological resorption in osteoporosis, inflammatory arthritis, and periodontitis (Figure 5).

Tanshinone, especially T-IIA, modulates this axis at three complementary levels. First, they temper the supply side by limiting RANKL production in osteoblasts and stromal cells. In LPS-challenged osteoblasts, T-IIA dose-dependently represses RANKL mRNA and protein while concomitantly increasing OPG expression, an effect that lowers the RANKL/OPG ratio by nearly 60% and is accompanied by inhibition of COX-2–derived prostaglandin E_2_, a potent upstream inducer of RANKL [99]. Similar shifts have been documented in periodontal tissues: local injection of T-IIA into the palatal gingiva (0.36 mg/d, 0.72 mg/d, and 1.44 mg/d) after orthodontic tooth movement (the maxillary first molar of rats was tracted to mesial movement using the anterior teeth as anchorage) raised OPG immunoreactivity and reduced RANKL expression, thereby enlarging the OPG/RANKL ratio and limiting relapse of tooth position [100]. Second, tanshinones control the essential side of bone remodeling by lowering the sensitivity of osteoclast precursors to RANKL [73,99]. Overexpression of c-Fos or NFATc1 rescues differentiation, positioning tanshinones upstream of the master transcriptional switch [99]. Third, in vivo studies demonstrate that these dual actions on RANKL availability and responsiveness translate into tangible skeletal protection. Systemic T-IIA curtails trabecular bone loss in ovariectomized mice, polyethylene–particle osteolysis, and LPS-induced calvarial destruction; histology from these models consistently shows reduced RANKL staining at osteoblast surfaces, elevated OPG, diminished phosphorylated NF-κB in osteoclast precursors, and fewer resorption lacunae [73,101].

In summary, tanshinones exert their bone-protective effects through the coordinated modulation of key signaling pathways involved in bone remodeling and inflammation. Central to their mechanism is the regulation of the RANKL–RANK–OPG axis, where they restore the balance between bone resorption and formation by reducing osteoclastogenesis while preserving physiological coupling. In parallel, tanshinones inhibit the PI3K/Akt and MAPK pathways, leading to decreased pro-inflammatory cytokine production and suppression of osteoclast differentiation and activity. These compounds also block MAPK phosphorylation, downregulate transcription factors such as c-Fos, AP-1, and NFATc1, and mitigate oxidative stress–induced signaling. Additionally, by targeting cathepsin K activity, tanshinones interfere with the enzymatic degradation of bone matrix. Collectively, these multifaceted actions highlight the therapeutic potential of tanshinones as dual-action agents capable of restoring bone homeostasis in inflammatory and osteolytic diseases.

## 4. Tanshinone and Osteoblast Signaling Pathways

### 4.1. Wnt/β-Catenin Signaling Pathway

The Wnt/β-catenin signaling pathway is pivotal in regulating osteoblast differentiation and bone formation. Activation of this pathway leads to the stabilization and nuclear translocation of β-catenin, which interacts with T-cell factor/lymphoid enhancer factor (TCF/LEF) transcription factors to promote the expression of osteogenic genes such as Runx2 and osteocalcin [102,103]. Tanshinone has been shown to enhance osteoblastogenesis by modulating the Wnt/β-catenin pathway. Specifically, tanshinone counteracts glucocorticoid-induced suppression of Wnt signaling by inhibiting the upregulation of Krüppel-like factor 15 (KLF15), a transcription factor that negatively regulates Wnt signaling [104]. This inhibition leads to the restoration of β-catenin levels and promotes osteogenic differentiation in mesenchymal stem cells (MSCs) under oxidative stress conditions. Furthermore, tanshinones modulation of the Wnt pathway contributes to the suppression of adipogenesis, thereby favoring osteoblast lineage commitment over adipocyte formation [47,104].

### 4.2. Bone Morphogenetic Protein (BMP) Signaling Pathway

BMP signaling, particularly through BMP-2, is essential for osteoblast differentiation and bone formation. Upon binding to its receptors, BMP-2 activates Smad proteins, which translocate to the nucleus to regulate the transcription of osteogenic genes [105]. A previous study investigated the synergistic effects of T-IIA on BMP signaling during osteoblast differentiation [106]. The main finding is that T-IIA significantly enhances BMP-2-induced osteogenic differentiation in C2C12 mesenchymal precursor cells. This enhancement occurs through the upregulation of osteogenic markers such as alkaline phosphatase, osteocalcin, and BMP isoforms (BMP-2, -4, -6, and -9). Mechanistically, T-IIA amplifies BMP-2-stimulated Smad1/5/8 phosphorylation and Runx2 activation, both of which are essential for osteoblast lineage commitment. This study also reveals that p38 MAPK plays a critical mediating role; T-IIA alone activates p38 and enhances BMP-2-dependent Smad signaling in a p38-dependent manner. Inhibition of p38 with SB202190 (a known p38 inhibitor) effectively blocks these enhancements, underscoring the role of p38 as a crucial signaling node in the crosstalk between tanshinone IIA and BMP-induced osteogenesis. These findings suggest that T-IIA promotes osteoblast differentiation through the p38–Smad–Runx2 axis, offering a potential therapeutic strategy for enhancing bone formation [106,107].

### 4.3. Fibroblast Growth Factor (FGF) Signaling Pathway

FGF signaling plays a complex role in bone biology, influencing both osteoblast proliferation and differentiation [108]. While FGF signaling is crucial for skeletal development, its overactivation can antagonize Wnt signaling, thereby inhibiting osteoblast differentiation [109]. Previous studies have shown the osteogenic potential of T-IIA on human periodontal ligament stem cells (hPDLSCs) [96]. This study demonstrates that T-IIA significantly enhances osteogenic differentiation, as evidenced by increased alkaline phosphatase activity, mineralized nodule formation, and elevated expression of osteogenic markers such as Runx2, osteopontin, and osteocalcin. Mechanistically, this study highlights the central role of the ERK1/2 MAPK pathway in mediating these effects, showing that T-IIA-induced Runx2 expression and subsequent osteogenesis are abolished when ERK1/2 is inhibited.

## 5. Preclinical Studies Investigating the Beneficial Effects of Tanshinone on Bone

### 5.1. Osteoporosis and Alveolar Bone Loss

Osteoporosis is a systemic skeletal disorder characterized by reduced bone mass and microarchitectural deterioration of bone tissue, leading to increased bone fragility and a heightened risk of fractures [2]. It occurs when bone resorption by osteoclasts outpaces bone formation by osteoblasts, resulting in porous and weakened bone structure. While commonly associated with postmenopausal women due to estrogen deficiency, osteoporosis can also affect men and may arise secondary to other medical conditions or medications [110]. Tanshinones have been evaluated in several rodent models of osteoporosis that mimic the clinical settings of estrogen deficiency and excess bone resorption. Across these studies, the compounds consistently preserve trabecular architecture, restrain osteoclast activity, and, in some contexts, support osteoblast or stromal cell function, as described below and in more detail in Table 2.

Wang et al. (2019) explores the therapeutic potential of T-IIA in a rat model of estrogen-deficiency-induced osteoporosis [111]. The findings of this study show prevention of alveolar-bone loss in ovariectomized rats, comprising higher bone volume, trabecular number and thickness, and lower trabecular separation than in untreated OVX controls. Mechanistically, T-IIA restored bone-marrow stromal-cell stemness and curtailed senescence by up-regulating phosphoglycerate dehydrogenase (PHGDH). This work suggests that T-IIA limits estrogen-deficiency-induced bone loss by rejuvenating stromal cells via epigenetic activation of PHGDH.

Daily T-IIA administration curtailed OVX-induced bone loss in a mouse model by blocking osteoclast differentiation and function [72]. In vitro, T-IIA dampened RANKL-triggered activation of NF-κB, MAPKs, and Akt; reduced TRAP, cathepsin K, and NFATc1 expression; inhibited actin-ring formation; and limited resorption pit area. Further study suggests that ten-week dosing with total tanshinone (200 mg kg per day) preserved lumbar and, to a lesser extent, tibial trabecular bone volume and thickness and lowered osteoclast surface [116]. Bone-formation indices and uterine weight were unchanged, indicating selective inhibition of resorption without estrogenic side effects. Thus, tanshinone acts as a bone-sparing, non-estrogenic anti-resorptive agent while maintaining bone formation [116].

Panwar et al. (2017) investigated the antiresorptive effects of STS in OVX mice, a model for postmenopausal osteoporosis [56]. STS selectively inhibited cathepsin K-mediated collagen degradation without affecting its peptide-cleaving activity, demonstrating efficacy in reducing bone resorption by osteoclasts while preserving bone formation. In OVX mice, STS treatment (40 mg/kg/day for 3 months) increased trabecular bone mineral density by 35%, reduced plasma CTx-1 levels by 20%, and elevated osteoblast activity without altering osteoclast numbers or exhibiting estrogenic effects. STS avoided side effects such as fibrosis by not disrupting TGF-β1 degradation in fibroblasts. This study highlights STS as a promising, selective antiresorptive agent with a favorable safety profile for OVX-mouse model management.

### 5.2. Diabetes Mellitus

Diabetes mellitus is a chronic metabolic disorder characterized by persistent hyperglycemia resulting from defects in insulin secretion, insulin action, or both. The condition is broadly classified into type 1 diabetes, which is caused by autoimmune destruction of pancreatic β-cells leading to absolute insulin deficiency, and type 2 diabetes, which involves insulin resistance combined with a relative insulin secretory defect [1]. Chronic hyperglycemia in diabetes is associated with long-term damage, dysfunction, and failure of various organs, especially the eyes, kidneys, nerves, heart, and blood vessels [1]. Importantly, diabetes adversely affects periodontal health by exacerbating inflammation, impairing immune response, and altering tissue repair mechanisms, which collectively accelerate the progression and severity of periodontitis [118].

Recently, Wei et al. (2024) investigated the protective effects of STS on vascular senescence in diabetic mice, focusing on the NFκB-NLRP3 inflammasome–catalase pathway [112]. This study found that STS treatment alleviated senescence in endothelial cells (ECs) and vascular smooth muscle cells (VSMCs) by preserving catalase (CAT) levels, reducing oxidative stress, and inhibiting NLRP3 inflammasome activation, which involves NLRP3 phosphorylation and dimer formation. Mechanistically, STS upregulated A20, an inhibitor of the NFκB pathway, thereby suppressing NLRP3 inflammasome activation and mitigating cellular senescence. These findings highlight STS as a potential therapeutic agent for diabetic vascular complications by targeting oxidative stress and inflammation.

Zhang et al. (2020) investigated the protective effects of T-IIA against osteoporosis in diabetic mice by targeting the renin–angiotensin system (RAS) [113]. In streptozotocin-induced diabetic mice, T-IIA treatment (10 and 30 mg/kg) significantly lowered serum and bone angiotensin II (ANG II) levels, improved trabecular bone mineral density, and enhanced bone microarchitecture, comparable to the renin inhibitor aliskiren. These results suggest T-IIA mitigates diabetic osteoporosis by suppressing renin-mediated ANG II production, highlighting its potential as a therapeutic agent for bone disorders in diabetes. Further research is needed to elucidate its molecular mechanisms and broader applications.

### 5.3. Rheumatoid Arthritis

Rheumatoid arthritis is a chronic, systemic autoimmune disease characterized by persistent synovial inflammation, leading to progressive joint destruction, pain, stiffness, and loss of function. The disease primarily affects the small joints of the hands and feet in a symmetrical pattern and is associated with systemic manifestations, including fatigue, anemia, and increased cardiovascular risk [3]. The pathogenesis of RA involves a complex interplay between genetic susceptibility, environmental factors, and immune dysregulation, resulting in the activation of autoreactive T and B cells, production of autoantibodies such as rheumatoid factor and anti-citrullinated protein antibodies, and secretion of pro-inflammatory cytokines [119]. These mediators contribute to synovial hyperplasia, pannus formation, cartilage degradation, and bone erosion.

The study by Wang et al. (2024) introduced an innovative injectable peptide-hydrogel system, pPNP + TIIA@PFS, designed to attenuate osteoarthritis (OA) progression by targeting senescent chondrocytes and promoting cartilage regeneration [114]. The hydrogel combines Klotho-expressing plasmid DNA (pPNP) and T-IIA to mitigate the senescent microenvironment, inhibit pro-inflammatory cytokine release, and block senescence signal transmission to healthy chondrocytes. Additionally, the hydrogel recruits BMSCs and directs their differentiation into chondrocytes, enhancing cartilage repair. In a rat OA model, the treatment significantly reduced osteophyte formation and cartilage degeneration while improving joint integrity. The findings highlight the potential of this dual-targeted approach, addressing both cellular senescence and cartilage regeneration, as a promising therapeutic strategy for OA.

A previous study investigated the therapeutic effects of T-IIA on OA by targeting angiogenesis in subchondral bone [117]. Using a monosodium iodoacetate (MIA)-induced OA mouse model, the researchers demonstrated that T-IIA administration significantly attenuated cartilage degeneration and normalized subchondral bone remodeling, as evidenced by micro-CT analysis showing improved bone volume/total volume (BV/TV) and subchondral bone plate thickness (SBP.Th). T-IIA also reduced osteoclast activity, as indicated by decreased TRAP-positive cells, and suppressed abnormal angiogenesis. Mechanistically, T-IIA diminished hypertrophic chondrocyte secretion of vascular endothelial growth factor A (VEGFA) and downregulated the VEGFA/VEGFR2/MAPK signaling pathway in endothelial cells, thereby reducing pathological angiogenesis. Additionally, Wang et al. (2019) investigated the therapeutic potential of T-I in OA, focusing on its anti-inflammatory and chondroprotective effects [115]. In vitro experiments using IL-1β-stimulated established cell line CHON-001 chondrocytes demonstrated that T-I significantly reduced apoptosis, suppressed inflammatory cytokines like TNF-α, and mitigated extracellular matrix degradation by upregulating collagen II while downregulating MMP-13. Additionally, T-I attenuated NF-κB activation and restored SOX11 expression, which is critical for cartilage maintenance. In a murine OA model, T-I treatment reduced cartilage destruction, synovitis, and subchondral bone thickening, as evidenced by improved OARSI scores and histological outcomes. These findings suggest that T-I may protect against OA progression by preserving chondrocyte function and inhibiting inflammatory pathways, highlighting its potential as a therapeutic agent for OA.

Panwar et al. (2024) investigated the dual anti-resorptive and anti-inflammatory effects of STS in a collagen-induced arthritis mouse model of rheumatoid arthritis [54]. STS significantly reduced bone and cartilage degradation by suppressing osteoclast activity and collagenolytic protease expression, as evidenced by decreased serum CTX I and CTX II levels and improved bone microarchitecture parameters (e.g., bone volume and trabecular thickness) in micro-CT analysis. Additionally, STS attenuated joint inflammation by inhibiting the NF-κB pathway, reducing phosphorylation of IκBα and p65, and downregulating pro-inflammatory cytokines (TNF-α, IL-1β, and IL-17) and immune cell infiltration (Th17 cells, macrophages, and neutrophils). In contrast, the active site-directed cathepsin K inhibitor odanacatib showed only anti-resorptive effects without impacting inflammation. These findings highlight STS as a promising therapeutic agent for RA, combining bone-protective and anti-inflammatory properties to mitigate joint destruction and disease progression.

### 5.4. Periodontal Disease

Only one study thus far has evaluated the beneficial effects of T-IIA and STS in an animal model of experimental periodontitis. Pavanelli et al. [44] investigated the anti-inflammatory and antiresorptive effects of T-IIA and STS in a murine model of ligature-induced periodontitis. The results demonstrated that both compounds significantly mitigated alveolar bone destruction by reducing osteoclast numbers and improving bone microarchitecture parameters, such as bone mineral density and trabecular thickness. Histological and immunohistochemical analyses revealed decreased inflammatory cell infiltration and downregulation of pro-inflammatory markers (IL-1β, IL-17, and MMP-13), corroborating the anti-inflammatory properties of tanshinones. Notably, STS specifically reduced cathepsin K mRNA levels, highlighting its selective inhibition of bone resorption. These findings suggest that T-IIA and STS exert dual protective effects by suppressing inflammation and osteoclast activity, positioning them as promising therapeutic agents for preventing periodontitis-associated bone loss [44].

### 5.5. Other Osteolytic Diseases

A previous study investigated the protective effects of T-IIA against polyethylene (PE) particle-induced osteolysis in a mouse calvarial model, a condition mimicking aseptic loosening in joint prostheses [101]. Using micro-CT and histomorphometric analyses, it was demonstrated that T-IIA significantly reduced bone resorption, osteoclast formation, and inflammatory responses by downregulating RANKL and OSCAR while upregulating OPG, thereby restoring the RANKL/OPG balance critical for bone remodeling. Moreover, the levels of bone resorption markers (CTX-1) were decreased, and increased OPG expression was observed in treated mice. Notably, T-IIA exhibited dose-dependent efficacy without causing hepatotoxicity or intestinal damage. These findings suggest its potential as a therapeutic agent to prevent wear particle-induced osteolysis and prosthetic loosening, though further studies are needed to validate long-term safety and efficacy in clinical settings.

Table 2 illustrates the experimental design and main findings of animal studies targeting bone inhibition using tanshinone treatment.

## 6. Concluding Remarks and Future Perspectives

Periodontitis is a chronic inflammatory disease marked by dysbiotic microbial communities, immune dysregulation, and progressive alveolar bone loss. Conventional treatments, including mechanical debridement and adjunctive antimicrobial therapies, frequently fall short of completely resolving inflammation or achieving regeneration of lost periodontal tissues. In this context, tanshinones offer a promising dual-action therapeutic approach by modulating both host immune responses and bone remodeling processes.

Tanshinones exert potent anti-inflammatory effects by inhibiting key signaling pathways such as NF-κB, MAPK, and the NLRP3 inflammasome, leading to reduced production of pro-inflammatory cytokines including IL-1β, TNF-α, and IL-17, all of which are central drivers of periodontal tissue destruction. Additionally, tanshinones attenuate osteoclast-mediated bone resorption by suppressing RANKL-induced osteoclastogenesis and cathepsin K activity while preserving the essential coupling between osteoclasts and osteoblasts necessary for bone homeostasis. Furthermore, they promote osteoblast differentiation and function through activation of osteogenic signaling pathways, including Wnt/β-catenin, BMP, and ERK/Runx2, thereby supporting the regeneration of periodontal structures.

Despite these promising biological effects, several challenges must be addressed to facilitate the clinical integration of tanshinones into periodontal therapy. One major hurdle is their limited pharmacokinetic profile. T-IIA, a principal compound in this class, exhibits poor oral bioavailability due to low aqueous solubility and extensive first-pass hepatic metabolism. Although STS improves solubility, further formulation advancements are necessary to achieve effective and localized drug delivery in periodontal tissues. Innovative delivery systems, such as injectable hydrogels, nanoparticles, and mucoadhesive films, could enhance site-specific retention and therapeutic efficacy. For example, T-IIA-loaded hydrogels, which have demonstrated success in preclinical arthritis models, could be adapted for intrapocket application in periodontal treatment. In this context, tanshinones could be incorporated into bioactive scaffolds or membranes to enhance alveolar bone repair. Alternatively, subgingival slow-release devices such as chips or microparticles may enable sustained drug release, paralleling existing delivery systems used for agents like chlorhexidine or doxycycline.

Safety and long-term outcomes also warrant careful evaluation. While tanshinones have demonstrated favorable safety profiles in short-term studies, their effects on long-term bone remodeling and systemic immune responses remain poorly characterized, especially in clinical studies. Bridging the gap between preclinical efficacy and clinical utility will require a multifaceted strategy centered on localized delivery, combination therapies, personalized medicine, and well-designed clinical trials.

Clinical translation will ultimately depend on robust human trials. Early-phase (Phase I/II) clinical studies should prioritize evaluation of safety, pharmacokinetics, and preliminary efficacy using endpoints such as probing depth reduction, clinical attachment gain, and radiographic bone density improvements. Incorporation of patient-reported outcomes, including pain levels and quality of life measures, as well as assessments of microbiological shifts in subgingival plaque, will provide a comprehensive understanding of therapeutic impact.

Beyond periodontitis, the therapeutic potential of tanshinones may extend to other inflammatory or osteolytic conditions in dentistry. In peri-implantitis, their dual anti-inflammatory and anti-resorptive actions may mitigate bone loss around dental implants. During orthodontic treatment, modulation of osteoclast activity by tanshinones could help reduce the risk of relapsing following tooth movement. In periapical disease, the effects of tanshinone might lead to beneficial effects on inhibiting apical bone loss. Moreover, their reported anti-tumor properties raise the possibility of inhibiting bone invasion in cases of oral squamous cell carcinoma.

In summary, tanshinones constitute a promising class of dual-action small molecules capable of simultaneously tempering inflammation, oxidative stress, and osteoclastogenesis while sparing or even stimulating bone formation. Their pharmacology addresses the central pathological axes of osteolytic disease and has yielded robust efficacy in a spectrum of preclinical models. While preclinical findings are compelling, further advances in drug delivery systems, safety evaluation, and clinical validation are essential to enable their clinical application. Bridging the translational gap will depend on demonstrating long-term safety and devising clinical trial paradigms that fully register their multimodal benefits. The ensuing review examines the molecular underpinnings, preclinical evidence, and translational prospects of tanshinone derivatives in osteolytic conditions, with the aim of informing rational bench-to-bedside development.

## Figures and Tables

**Table 1:** Summary of tanshinone types and their biological properties.

Tanshinone Type	Biological Effects	Key Properties
Tanshinone IIA	- Anti-inflammatory: Inhibits NF-κB, TNF-α, IL-6, and IL-1β. - Osteoprotective: Reduces osteoclastogenesis via RANKL inhibition. - Antioxidant: Scavenges ROS, protecting bone cells.	Lipophilic diterpenoid quinone; suppresses inflammatory cytokines (IL-1β, IL-17); modulates signaling pathways; and improves bone density and architecture in vivo.
Sodium Tanshinone IIA Sulfonate (STS)	- Selective cathepsin K ectosteric inhibitor. - Antiresorptive properties without affecting osteoclast metabolism. - Anti-inflammatory and anti-oxidative properties inhibit vascular senescence via NLRP3 inflammasome suppression.	Water-soluble derivative of T-IIA; protects endothelial and smooth muscle cells from oxidative stress; and downregulates cathepsin K. Binds ectosteric site on CatK; reversible inhibition; reduces inflammatory cytokines and osteoclast activity; and avoids side effects of active-site inhibitors.
Tanshinone I	- Modulates inflammation: Reduces COX-2 and iNOS. - Suppresses osteoclastogenesis via downregulation of c-Fos and NFATc1. - Antioxidant effects.	Lipophilic diterpenoid; modulates NF-κB and apoptosis pathways; and protects cartilage in vitro and in vivo models.
Cryptotanshinone	- Strong anti-inflammatory properties: Blocks NF-κB and STAT3 signaling. - Osteoclast inhibition: Downregulates RANKL-induced TRAP+ cells. - Promotes osteoblast activity.	Diterpenoid quinone; modulates inflammation and osteoclast activity; and exact mechanisms less studied independently.
dihydrotanshinone I	- Potent anti-resorptive: Stronger osteoclast inhibitor. - Induces apoptosis in mature osteoclasts. - Antimicrobial and anti-angiogenic properties.	Principal lipophilic phenanthraquinone compound found in Salvia miltiorrhiza. It has a broad range of biological roles, including antibacterial and anti-inflammatory, antioxidant, and regulation of immune cells.

**Table 2:** Detailed summary of tanshinone effects in preclinical models of osteolytic diseases.

Authors (Year)	Tanshinone Type	Experimental Design	Animal Model/Methodology	Main Results
Wang et al. (2019) [111]	T-IIA	Investigate the effects and molecular mechanisms of tanshinone on osteoporosis. T-IIA was administered by tail vein injection at a dose of 10 mg/kg daily for two weeks.	Osteoporosis was induced by bilateral ovariectomy (OVX) in adult female rats treated with or without T-IIA. Trabecular bone structure was assessed by micro-CT, and levels of age-related genes were measured by mRNA.	T-IIA preserved bone volume and microarchitecture, enhanced trabecular number and thickness, and reduced trabecular separation. Mechanistically, it rejuvenated stromal cells by upregulating PHGDH, countering estrogen-deficiency-induced senescence. Tanshinone potently suppresses OVX-induced osteoporosis and BMSC senescence through upregulation of PHGDH.
Panwar et al. (2017) [56]	STS	In vitro and in vivo study using human and mouse osteoclasts and OVX mice. Adult C57BL/6 mice received 40 mg/kg/d STS by oral gavage for 3 months.	STS was tested for collagen degradation inhibition, osteoclast activity, bone resorption, and in vivo bone parameters in OVX mice.	STS selectively inhibited collagen degradation and suppressed bone resorption in human and mouse osteoclasts without affecting osteoclastogenesis or metabolism. In OVX mice, 3-month STS treatment reduced plasma CTx-1 by 20%, increased osteoblasts and P1NP (~28%), and improved femoral BMD by 35%.
Wei et al. (2024) [112]	STS	Examined vascular senescence in diabetic mice treated with STS. Focused on the NFκB–NLRP3–catalase axis. In vivo (diabetic mice) and in vitro (primary ECs and VSMCs under high glucose) study. Diabetic mice were treated with intravenous injections of 10 mg/kg/day STS for 90 days.	Diabetic mice and primary vascular cells were treated with STS and transfected with NLRP3 and A20 overexpression/knockout plasmids; senescence markers and signaling pathways were assessed.	STS reduced vascular senescence in diabetic mice by maintaining catalase levels, improving vascular relaxation, and lowering oxidative stress and senescence markers (p21, SA-β-gal, and collagen). Mechanistically, STS inhibited NLRP3 phosphorylation, dimerization, and inflammasome activation while preserving A20 and CAT expression and suppressing NFκB signaling in ECs and VSMCs under high glucose.
Zhang et al. (2020) [113]	T-IIA	Assessed T-IIA’s impact on diabetic osteoporosis through RAS modulation. In vitro (renin-expressing HEK-293 cells) and in vivo (STZ-induced diabetic mice) study.	T-IIA was screened for renin inhibition in engineered HEK-293 cells; diabetic C57BL/6 mice were treated with T-IIA (10 or 30 mg/kg) or aliskiren. ANG II levels and bone parameters were assessed.	T-IIA inhibited renin activity and reduced ANG II expression in vitro. In diabetic mice, it decreased serum ANG II levels and expression in bone and improved trabecular bone mineral density and structure in the tibia and femur. Findings suggest T-IIA as a potential renin inhibitor with osteoprotective effects in diabetic osteoporosis.
Wang et al. (2024) [114]	T-IIA	Developed a peptide-hydrogel (pPNP+TIIA@PFS) for OA therapy targeting senescent chondrocytes using in vitro and in vivo (surgically induced osteoarthritis in rats) models. The pPNP + TIIA@PFS was injected into the rat knee joint to assess its therapeutic efficacy against OA.	Developed an injectable peptide hydrogel (pPNP + TIIA@PFS) delivering T-IIA and DNA-loaded nanoparticles; evaluated effects on chondrocyte senescence, cartilage regeneration, and OA progression	pPNP + TIIA@PFS increased anti-aging protein Klotho, blocked senescence signaling, reduced chondrocyte senescence, and improved cartilage integrity. It recruited bone mesenchymal stem cells and promoted chondrogenesis. In OA rats, it reduced osteophyte formation and cartilage degeneration, indicating therapeutic potential for OA.
Pavanelli et al. (2025) [44]	T-IIA and STS	Explored T-IIA and STS effects in a murine model of ligature-induced periodontitis in C57BL/6 mice.	C57BL/6J mice assigned to control, periodontitis, T-IIA, and STS groups; treated with 40 mg/kg tanshinones via oral gavage for 10 days; assessed via micro-CT, histology, immunohistochemistry, and RT-qPCR.	Both T-IIA and STS reduced inflammatory cell infiltration, increased fibroblast count, prevented alveolar bone loss, improved bone architecture and mineral density, reduced osteoclast numbers, and suppressed IL-1β, IL-17, and MMP-13. STS significantly reduced cathepsin K expression. Findings support their anti-inflammatory and antiresorptive potential in periodontitis.
Panwar et al. (2024) [54]	STS	Evaluated anti-inflammatory and anti-resorptive actions of STS in collagen-induced arthritis (CIA). CIA was induced by injecting 200 μg of bovine type II collagen emulsified with complete Freund adjuvant with 0.5 mg/mL of *M. tuberculosis* into the tail base, followed by 200 μg of immunization with bovine type II collagen and IFA. STS (40 mg/kg/d) was mixed with food powder (3 g chow per mouse).	Compared STS with active site inhibitor odanacatib (ODN); assessed joint pathology, cytokines, osteoclasts, and NF-κB signaling using histopathology, flow cytometry, and biochemical assays.	STS reduced immune cell infiltration, inflammatory cytokines (incl. IL-17), Th17 cells, and osteoclasts in joints. It selectively inhibited CatK collagenolytic activity via oligomerization blockade and suppressed the NF-κB pathway by inhibiting IκBα phosphorylation. ODN showed only antiresorptive activity without anti-inflammatory effects. T06 demonstrated dual therapeutic action in RA.
Wang et al. (2019) [115]	T-I	Investigated anti-inflammatory and cartilage-protective effects in vitro (IL-1β-induced OA model in CHON-001 cells) and in vivo using an anterior cruciate ligament transection mouse model (ACLT-induced OA in mice). Mice were treated with T-I via intraperitoneal injection once daily for 8 weeks after surgery using 10 mg/kg or 30 mg/kg T-I.	CHON-001 cells pretreated with T-I (20 μM) and stimulated with IL-1β; mice treated with T-I (10 or 30 mg/kg) for 8 weeks post-ACLT. Cell viability, apoptosis, ECM degradation, and inflammation were assessed via CCK-8, flow cytometry, Western blot, and histological staining.	T-I reduced IL-1β-induced apoptosis, preserved collagen II and aggrecan, suppressed MMP-13, cleaved caspase 1, Gasdermin D, and p-NF-κB, and restored SOX11 expression in vitro. In vivo, it alleviated cartilage degradation, synovitis, and subchondral bone loss and reduced OARSI scores, suggesting therapeutic potential in OA.
Panwar et al. (2018) [55]	STS	Screened tanshinone derivatives for cathepsin K (CatK) inhibition. In vitro enzymatic and cell-based assays using human osteoclasts.	Screened 31 tanshinones from Salvia miltiorrhiza for CatK inhibition; assessed collagen degradation, bone resorption, cell viability, osteoclastogenesis, reversibility, and binding sites via enzymatic assays, SEM, mechanical testing, and molecular docking.	Twelve tanshinones showed selective anti-collagenase activity without affecting non-collagenous substrates. Six compounds strongly inhibited osteoclast-mediated bone resorption (IC50 < 500 nM) without impacting cell viability or osteoclastogenesis. The core pharmacophore was identified as a three-ring structure with para- or ortho-quinone. Findings support ectosteric CatK inhibition as a safer therapeutic approach.
Cheng et al. (2018) [72]	T-IIA	In vivo study using ovariectomized (OVX) C57BL/6 mice to model postmenopausal osteoporosis; in vitro study using bone marrow-derived macrophages (BMMs) to evaluate osteoclastogenesis. T-IIA (10 mg/kg) was given by intraperitoneal injection daily for 6 weeks.	OVX mice were treated with T-IIA to assess bone loss prevention. In vitro, BMMs were stimulated with RANKL in the presence or absence of T-IIA to evaluate osteoclast formation. Western blot, TRAP staining, and immunofluorescence were used to analyze signaling pathways and osteoclast markers.	T-IIA prevented bone loss in OVX mice and inhibited RANKL-induced osteoclast differentiation in vitro. It blocked NF-κB, Akt, and MAPK signaling pathways, reducing phosphorylation of IκB, ERK, p38, and Akt, and nuclear NF-κB p65 translocation. Osteoclast-related gene expression was also decreased. Suggests therapeutic potential in postmenopausal osteoporosis.
Cui et al. (2004) [116]	Total tanshinone (containing 17% of T-IIA and 8% of cryptotanshinone)	In vivo study using OVX Sprague–Dawley female rats: four groups including sham-operated control, OVX + vehicle, OVX + total tanshinone, and OVX + 17α-ethynylestradiol (positive control). Treatments started 1 day post-OVX and continued daily for 10 weeks.	Rats were administered total tanshinone (200 mg/kg/day, equivalent doses of T-IIA and cryptotanshinone), vehicle, or estrogen. Bone histomorphometry of lumbar vertebrae (LV4) and proximal tibial metaphyses (PTM) was performed.	Tanshinone prevented OVX-induced decreases in trabecular bone volume and number and increases in osteoclast surface in LV4 and partially protected PTM. Tanshinone increased trabecular thickness and did not affect mineralizing surface, body, or uterine weight. Estrogen increased bone volume but decreased mineralizing surface and increased uterine weight. Tanshinone prevented bone loss by inhibiting bone resorption without estrogenic side effects.
Li et al. (2024) [117]	T-IIA	In vivo study using a monosodium iodoacetate (MIA)-induced osteoarthritis (OA) mouse model; in vitro assays with primary CD31^hi^Emcn^hi^ endothelial cells T-IIA (20 mg/kg) was administered daily via intragastric administration for 2 weeks.	MIA was injected to induce OA in mice, followed by T-IIA treatment to evaluate effects on cartilage degeneration, subchondral bone remodeling, and angiogenesis. Endothelial cell angiogenesis assays and hypertrophic chondrocyte culture supernatant were used to study mechanisms.	TIIA attenuated cartilage degeneration, normalized subchondral bone remodeling, and suppressed aberrant angiogenesis in vivo. It reduced hypertrophic chondrocyte numbers and VEGFA secretion, inhibited tube formation of endothelial cells, and downregulated VEGFR2 and MAPK signaling. Results highlight TIIA as a potential anti-angiogenic therapeutic agent for OA.
Yao et al. (2018) [101]	T-IIA	In vivo study using a polyethylene (PE) particle-induced osteolysis mouse calvarial model: C57BL/6J male mice divided into sham, PE+PBS, PE+low-dose T- IIA, and PE+high-dose T-IIA groups T-IIA (1 or 2 µg/g) was locally injected into the mouse skull for 21 days.	PE particles implanted on calvaria to induce osteolysis; mice treated with T-IIA (1 or 2 µg/g) or PBS for 21 days. Bone resorption assessed by micro-CT and histomorphometry; osteoclast activity markers (OSCAR and CTX-1) and OPG measured by ELISA.	T-IIA dose-dependently reduced PE particle-induced bone resorption and osteoclast formation/activity. It decreased OSCAR and CTX-1 levels and increased OPG expression, protecting bone around implants. Suggests potential to prevent aseptic loosening post-joint replacement.

## Data Availability

No new data were generated in this study.
